# Walking Uphill Aggravates Dyspnea and Dynamic Hyperinflation at Equivalent Oxygen Uptake in COPD Patients

**DOI:** 10.3390/jcm15124601

**Published:** 2026-06-13

**Authors:** Ronen Reuveny, Amit Yaniv, Einat Kodesh, Tal Krasovsky, Arie Rotstein, Ariela Velner, Michael J. Segel

**Affiliations:** 1 Pulmonary Institute, Sheba Medical Center, Tel-HaShomer, Ramat Gan 5266202, Israel; ronen.reuveny@sheba.health.gov.il (R.R.); trainprivate@gmail.com (A.Y.); ariela.velner@sheba.health.gov.il (A.V.); 2 Physical Therapy Department, Faculty of Social Welfare and Health Sciences, University of Haifa, Haifa 3103301, Israel; tkrasovsk@univ.haifa.ac.il; 3 Dina Recanati School of Medicine, Reichman University, Herzliya 4610101, Israel; 4 Zinman College of Physical Education and Sport Sciences, Levinsky-Wingate Academic College, Wingate Campus, Netanya 4290200, Israel; rotstein@l-w.ac.il; 5 Pediatric Rehabilitation Department, The Edmond and Lily Safra Children’s Hospital, Sheba Medical Center, Ramat Gan 5266202, Israel; 6 Gray Faculty of Medicine, Tel-Aviv University, Tel Aviv 6997801, Israel

**Keywords:** COPD, exertional dyspnea, hyperinflation, treadmill, cardiopulmonary exercise testing, incline, uphill

## Abstract

**Background/Objectives**: COPD patients often complain of severe dyspnea when walking uphill, even up a mild incline. This study aimed to determine whether the dyspnea experienced during uphill walking is disproportionate to the increased mechanical work required to overcome gravity. **Methods**: Fourteen COPD patients (FEV_1_ 49 ± 11% predicted) and nine healthy participants performed three symptom-limited exercise tests on a treadmill, each at a fixed grade: 1%, 2.5%, and 4% for COPD patients; and 1%, 3%, and 5% for healthy participants. Treadmill speed was increased stepwise (3 min/stage). Inspiratory capacity (IC) maneuvers were performed during the last minute of each stage. Borg dyspnea scores (0–10) at the different inclines were compared at a uniform level of oxygen uptake (iso-$\dot{V}$O_2_). **Results**: Borg dyspnea scores by COPD patients at the highest iso-$\dot{V}$O_2_ attained were significantly higher at 4% treadmill grade compared to 2.5% and compared to 1% grade (7 ± 2 vs. 5 ± 2 vs. 5 ± 2, respectively; *p* < 0.001 for 4% vs. 1% grade, *p* < 0.005 for 4% vs. 2.5%). Dynamic hyperinflation worsened with grade, as reflected by decrease in inspiratory reserve volume (IRV) at the highest common iso-$\dot{V}$O_2_ attained: 798 ± 336 mL at 1% grade vs. 698 ± 325 mL at 2.5% (*p* < 0.004) vs. 564 ± 350 mL at 4% (*p* < 0.002 for 4% vs. 1%; *p* < 0.004 for 4% vs. 2.5%). In contrast, healthy participants showed no significant grade-dependent differences in dyspnea or IRV at iso-$\dot{V}$O_2_. **Conclusions**: Walking uphill in itself increases breathlessness of COPD subjects at iso-$\dot{V}$O_2_, suggesting that the increased dyspnea cannot be explained simply by the increased work. This phenomenon may be related to dynamic hyperinflation, which is worse at steeper inclines.

## 1. Introduction

Chronic obstructive pulmonary disease (COPD) is a major cause of morbidity and mortality worldwide. The physiological hallmark of COPD is persistent airflow obstruction—airway narrowing and reduced lung elastic recoil lead to dynamic airway closure and expiratory flow limitation [1]. 

Dyspnea on exertion is the primary symptom of COPD, limiting exercise and often leading to avoidance of physical activity, with consequent skeletal muscle and cardiovascular deconditioning [2]. As ventilatory demand increases during exertion, expiratory flow limitation forces the COPD patient to breathe at higher lung volumes, a phenomenon known as dynamic hyperinflation (DH) [3]. However, while breathing at higher operating volumes increases expiratory flow, it also requires the COPD patient to overcome the flatter portion of the respiratory compliance curve, thereby increasing the work of breathing. This mechanical constraint is associated with a marked increase in dyspnea, particularly when the inspiratory reserve volume reaches a critical threshold (below approximately 700 mL) [2,4]. DH has been shown to limit exercise even in patients with spirometrically mild COPD [5,6]. DH can be assessed during cardiopulmonary exercise testing by performing inspiratory capacity maneuvers and tidal flow-volume loop analysis [4,7,8]. 

COPD patients frequently complain that dyspnea worsens markedly when walking uphill, even when the incline (grade) is very mild. A study that investigated the dyspnea response to changing treadmill grade in COPD patients during a 2-min dyspnea challenge test found that the Borg dyspnea score (BDS) increased significantly with as little as a 2% increase in the treadmill grade [9]. Although any increase in incline increases the work required to overcome gravity, the disproportionate dyspnea observed with such small changes in grade suggests that factors beyond the additional metabolic demand may contribute to this response. Specifically, we hypothesized that walking uphill might exacerbate DH. We therefore designed the current study to compare dyspnea and DH across different treadmill grades at a uniform level of oxygen consumption (iso-$\dot{V}$O_2_), in order to neutralize the effect of increased work against gravity, enabling us to determine whether walking uphill, in itself, increases respiratory constraint and the severity of dyspnea.

## 2. Materials and Methods

### 2.1. Study Design

Single-center, random-order cross-over study.

### 2.2. Participants

Sixteen clinically stable COPD subjects (COPD group) and 10 healthy subjects (healthy group) were included in the study. Recruitment was between 1 August 2018 and 31 July 2020. COPD patients were recruited from the pulmonary clinic at our institution. Healthy subjects were recruited among colleagues. Inclusion criteria for the COPD group were (1) post-bronchodilator forced expiratory volume in one second: forced vital capacity ratio (FEV_1_: FVC) <0.70; (2) post-bronchodilator FEV_1_ < 80% of predicted. Patients experiencing respiratory exacerbation within 1 month of enrollment or during the study period, or with cardiovascular, orthopedic, neuromuscular or other comorbid conditions that might limit exercise capacity were excluded from both groups. Healthy subjects were non-smokers and were required to have normal lung function. The Institutional Ethics Committee for Human Medical Research approved the study (No. 5036-18-SMC). All subjects provided written informed consent. Individual participants completed all evaluations during three visits within no more than 4 weeks. No COPD exacerbations occurred during the study. 

### 2.3. Pulmonary Function Tests

Spirometry, body plethysmography and single breath carbon monoxide diffusing capacity (DLCO) were performed during the first visit according to ATS-ERS guidelines. Predicted values for lung function were calculated from Global Lung Initiative (GLI) normal values [10]. Maximal voluntary ventilation (MVV, L/min) was measured over 12 s. MVV was also calculated for each patient as FEV_1_ (L) multiplied by 40 [8,11]. To calculate the breathing reserve (BR) we used the higher of the measured (pre- and post-exercise) and calculated MVV values. Spirometry was also performed before and after the completion of each exercise test, to ensure stability of the respiratory impairment, and for use in tidal flow limitation analysis (see below), respectively.

### 2.4. Cardiopulmonary Exercise Testing 

Symptom-limited exercise tests were performed on a treadmill (TMX428, TrackMaster, Newton, KS, USA). Subjects breathed through a face mask connected to a metabolic cart (CareFusion, Wurzburg, Germany). All subjects performed three exercise tests (on separate visits), each at different fixed treadmill grade: 1%, 2.5% and 4% for the COPD group and 1%, 3% and 5% for the healthy group. Because patients with COPD were expected to have substantially lower exercise tolerance than healthy participants, treadmill grades and speeds were selected a priori to ensure that participants in both groups could complete multiple exercise stages, including during the test at the highest incline planned. Tests were performed in a randomized order determined by lottery. A minimum interval of 3 days was maintained between tests. Although no formal assessment of carryover effects was performed, the ≥3-day recovery interval was intended to minimize their potential influence. Subjects were blinded to the treadmill grade and speed. Treadmill speed was increased every 3 min. For severe COPD subjects with FEV_1_ < 50% predicted (nine subjects), the initial speed was 2.0 km/h and was increased by 0.3 km/h at each stage. For COPD subjects with FEV_1_ > 50% predicted (five subjects) the initial speed was 2.5 km/h and was increased by 0.5 km/h at each stage. For healthy subjects, the initial speed was 3.0 km/h and increased by 1.0 km/h each stage. Exercise tests were continued until volitional exhaustion. Oxygen uptake ($\dot{V}$O_2_), carbon dioxide output ($\dot{V}$CO_2_), heart rate (HR), 12-lead ECG, and oxygen saturation (SpO_2_%) were continuously monitored throughout the test. Respiratory exchange ratio (RER), tidal volume (V_T_), breathing frequency (B_f_), minute ventilation ($\dot{V}$_E_) and end-tidal partial pressure of CO_2_ (PetCO_2_) were recorded continuously. The modified Borg scale [7] was used to rate perceived dyspnea (“breathing discomfort”) and leg fatigue at the end of each stage during exercise. To compare the development of dyspnea and dynamic hyperinflation throughout exercise, dyspnea ratings and inspiratory reserve volume (IRV) were also evaluated at standardized relative exercise intensities corresponding to 60%, 70%, 80%, 90%, and 95% of each participant’s peak $\dot{V}$O_2_. Peak $\dot{V}$O_2_ was determined by averaging the last 30 s of breath-by-breath values. Peak $\dot{V}$O_2_ was reported relative to body mass (mL·kg^−1^·min^−1^), and, where indicated, as a percentage of the predicted value (% predicted) using predicted values of Kaminsky et al. [12]. Gas exchange threshold (GET) was determined manually using the V-slope method [13]. Criteria for maximal effort were respiratory exchange ratio (RER) > 1.1 or HR > 90% predicted in healthy participants, and/or evidence of ventilatory limitation in COPD subjects.

### 2.5. Lung Volume Measurements During Exercise

Spirometry and MVV measurements were performed before each exercise test. To familiarize subjects with the maneuver, inspiratory capacity (IC) maneuvers were practiced prior to the exercise tests, while the subject was standing still. During the exercise tests, IC maneuvers were performed at rest, twice during the final 40 sec of each exercise stage, and after 2 min of recovery. As previously described, ref. [14], subjects were first asked to breathe normally in order to capture a tidal flow volume loop (tFVL). The attending physiologist monitored the spirogram to ensure stability of the tidal volume and then instructed the subject to inhale fully after a normal exhalation (i.e., from end-expiratory lung volume (EELV) to total lung capacity (TLC)), then to passively exhale to EELV. During the IC maneuver subjects were verbally encouraged to inspire maximally, and then to exhale passively, without holding their breath [15]. Dynamic hyperinflation was determined to be present if IC decreased more than 150 mL during exercise [4]. The inspiratory reserve volume (IRV) was calculated by subtracting the end inspiratory lung volume (EILV; EILV = EELV + TV) from TLC (IRV = TLC − EILV) [8]. A critical increase in the elastic load of breathing was determined to be present if IRV during exercise was less than 700 mL or if EILV/TLC was greater than 0.9 [4]. 

### 2.6. Linear Interpolation 

To compare physiological responses at a uniform metabolic load, parameters were evaluated at the same oxygen uptake (iso-$\dot{V}$O_2_). For each participant, iso-$\dot{V}$O_2_ was defined as the lowest peak $\dot{V}$O_2_ achieved across the three treadmill tests. Values of the study variables at iso-$\dot{V}$O_2_ were estimated separately for each treadmill condition using linear interpolation between the two measured data points immediately above and below the selected iso-$\dot{V}$O_2_ value. No extrapolation beyond the measured data was performed. This procedure generated one iso-$\dot{V}$O_2_ value per participant for each treadmill incline, which was subsequently analyzed using repeated-measures statistical methods. An example of the interpolation procedure is provided in Supplementary Figure S1.

### 2.7. Gait Parameters

Gait parameters were recorded during exercise tests using an OptoGait system (MicroGate, Bolzano, Italy) mounted on the treadmill. Individual step data were analyzed using customized code in Matlab (version 25, Natick, MA, USA). Gait data were extracted during one minute of recorded walking, 40 sec after the onset of each speed change. In order to compare across inclines, and given the dependence of gait parameters on speed, we extracted gait parameters for the highest common speed which was present in all incline conditions (iso-speed). Means and coefficients of variation (CV) for step length and step time were used. Outlier steps were removed if they exceeded three scaled median absolute deviations. 

### 2.8. Statistical Analysis

Normality distribution was assessed using the Shapiro–Wilk test. Several variables showed significant deviations from normality, particularly $\dot{V}$O_2_/kg and gait variability measures such as step length CV, step time CV, and speed CV. Given these violations and the small sample size, the assumption of normality was not considered sufficiently met. Therefore, non-parametric analyses were used, including the Mann–Whitney U-test for comparisons between COPD and healthy subjects. The non-parametric repeated measures Friedman test with post-hoc Dunn test were used to compare the physiological responses between the three different inclines at iso-$\dot{V}$O_2._ For gait parameters, no between-group comparisons were made due to differences in gait speeds between groups. To compare gait parameters between inclines, Friedman’s test was used with post-hoc Dunn’s test. A *p*-value < 0.05 was considered statistically significant. In all cases, post hoc tests were performed only when the Friedman test was significant, and *p*-values were adjusted for multiple comparisons using a Bonferroni correction (*p* < 0.025). Data were analyzed using SPSS (v25.0, SPSS Inc., Chicago, IL, USA) or GraphPad Prism (version 4.0, GraphPad Software, La Jolla, CA, USA).

## 3. Results

Twenty-six subjects were enrolled in this study. Three subjects (two COPD subjects and one healthy subject) did not complete the study because the effort performed in the first exercise test was judged to be submaximal. No COPD exacerbations occurred during the study. Overall, 23 subjects completed all tests according to the study protocol and were included in the final analysis: 14 COPD subjects (four female) and nine healthy subjects (two female). Gait parameters were analyzed for 12 COPD and five healthy control subjects, due to technical issues with equipment integration. The resulting missing data were assumed to be missing at random. 

Baseline characteristics of the study population, including lung function, are shown in Table 1. The groups were well-matched for sex, age, height, weight, and BMI. COPD subjects had smoking histories of 15–100 pack-years (median: 50; two were active smokers) and had varying degrees of airway obstruction (FEV_1_ 30–73% predicted), air trapping (RV/TLC 0.50–0.69) and hyperinflation (TLC 80–145% predicted). COPD subjects had normal to severely reduced DLCO (37–107% predicted). The healthy group were all non-smokers and had normal lung function. 

### 3.1. Peak Exercise Capacity

Results at peak exercise, determined for each subject as the highest peak $\dot{V}$O_2_ achieved during the three exercise tests, are shown in Table 2. As expected, peak $\dot{V}$O_2_ was significantly lower in COPD subjects compared to healthy subjects (*p* < 0.009). Minute ventilation ($\dot{V}$_E_) and tidal volume (V_T_) at peak exercise were significantly reduced in COPD subjects compare to healthy subjects as was breathing reserve (BR%). Oxygen saturation (SPO_2_%) at peak exercise was significantly reduced in COPD subjects compared to healthy subjects. There were no significant differences in the ventilatory equivalents for CO_2_ or O_2_ ($\dot{V}$_E_/$\dot{V}$CO_2_ and $\dot{V}$_E_/$\dot{V}$O_2_, respectively) at the gas exchange threshold (GET) or for end-tidal CO_2_ (PetCO_2_) at peak exercise. 

### 3.2. Dyspnea at Iso-$\dot{V}$O_2_ Increases with Treadmill Grade in COPD Subjects 

**Figure 1:**
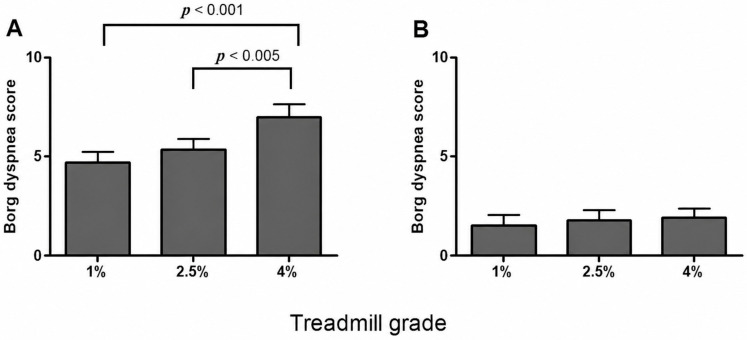
Dyspnea rating (Borg scale) at iso-$\dot{V}$O_2_ compared between treadmill grades in COPD subjects (**A**) and healthy participants (**B**). *p*-values are from Dunn’s post-hoc tests, performed only when the Friedman test showed significance.

Dyspnea ratings at iso-$\dot{V}$O_2_ compared between three grades of incline in COPD subjects and healthy subjects are shown in Figure 1. In COPD subjects, dyspnea ratings at iso-$\dot{V}$O_2_ were significantly higher at 4% treadmill grade compared to 2.5% grade (*p* < 0.01) and 1% grade (*p* < 0.001) (Figure 1A). In healthy subjects, there was no significant difference between treadmill grades in dyspnea score at iso-$\dot{V}$O_2_ (Figure 1B). These results suggest that, specifically in the COPD group, the incline (treadmill grade) in itself increases dyspnea, beyond the effect of the increased work attributable to the increase in gravitation. 

### 3.3. Dynamic Hyperinflation Increases with Treadmill Grade in COPD Subjects 

**Figure 2:**
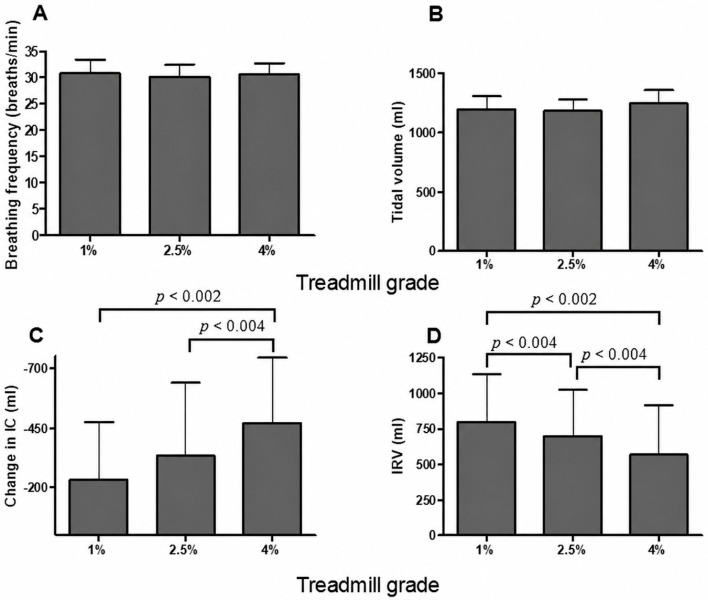
Effect of treadmill grade (1%, 2.5%, and 4%) on ventilatory mechanics in the COPD group at iso-$\dot{V}$O_2_. (**A**) Breathing frequency. (**B**) Tidal volume. (**C**) Change in inspiratory capacity (IC). (**D**) Inspiratory reserve volume (IRV). *p*-values are from Dunn’s post-hoc tests, performed only when the Friedman test showed significance.

In order to understand the mechanism of the exacerbation of dyspnea by increased treadmill grade we studied the effect of grade on breathing patterns. Dynamic lung volumes and gas exchange in the COPD group during exercise at different grades are shown in Table 3. Treadmill grade did not affect $\dot{V}$_E_, SPO_2_%, breathing frequency or tidal volume at iso-$\dot{V}$O_2_ (Figure 2A,B). $\dot{V}$_E_/$\dot{V}$CO_2_, RER and PetCO_2_ were also unaffected by treadmill grade (Table 3). However, COPD subjects developed worse dynamic hyperinflation as treadmill grade increased, manifest as a greater dynamic decrease in IC and IRV (Figure 2C and Figure 2D, respectively). In contrast, in the healthy group, IC increased modestly during exercise, and there was no difference between the treadmill grades (A detailed description and graphical example of the interpolation procedure are provided in Supplementary S1, Figure S1 and Table S1). 

### 3.4. Dyspnea and IRV at Exercise Intensities Corresponding to % $\dot{V}$O_2_ Peak

**Figure 3:**
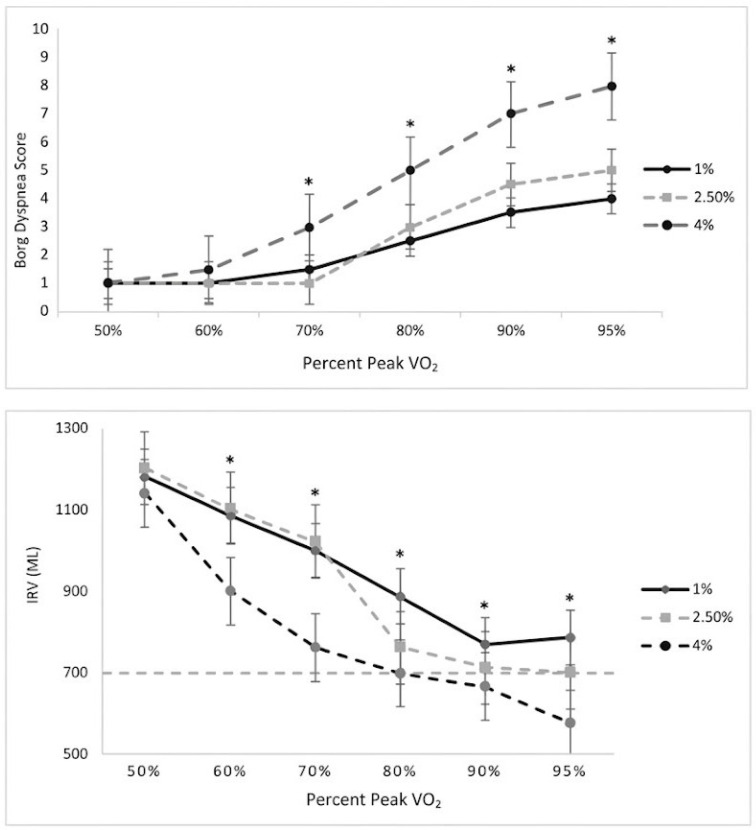
Dyspnea score (**upper panel**) and inspiratory reserve volume (IRV) (**lower panel**) relative to individual %$\dot{V}$O_2_ peak (peak oxygen consumption) in COPD patients. * *p* < 0.05 (Dunn’s post-hoc tests, performed only when the Friedman test showed significance).

Dyspnea ratings as a function of $\dot{V}$O_2_ (expressed as % of individual peak $\dot{V}$O_2_) were compared between treadmill gradients (Figure 3). Dyspnea score at 4% began to rise relative to the 1% grade at 60% of peak $\dot{V}$O_2_, becoming statistically significant by 70% of peak $\dot{V}$O_2_ (Figure 3, upper panel). Changes in IRV were significantly greater at 4% than at 1% grade, already at 60% of peak $\dot{V}$O_2_ (Figure 3, lower panel). Although the differences between the 2.5% and 1% grades did not reach statistical significance, a similar trend of increased dyspnea and greater hyperinflation (a greater decrease in IRV) was observed starting at 80% of peak $\dot{V}$O_2_. In the healthy group, no differences were found between the treadmill grades in dyspnea score or IRV during all exercise intensities up to 90% of $\dot{V}$O_2_.

### 3.5. Gait Parameters Across Treadmill Grades

When controlling for gait speed, a significant increase in variability of step time was associated with increased grade in the COPD group only (χ^2^ =13.2, *p* = 0.001) (Supplementary Table S2). Specifically, the 4% (median CV: 3.6% (IQR 3.0–5.1); Z = −2.51, *p* = 0.012) and 2.5% (median CV: 4.0% (IQR 2.7–4.6); Z = −2.9, *p* = 0.004) grades were associated with higher variability of step time compared to the 1% grade (median CV: 4.0% (IQR 2.5–4.7)). No other significant differences were found. 

## 4. Discussion 

The present study demonstrates that, at a given metabolic load (iso-$\dot{V}$O_2_), walking uphill exacerbates dyspnea in patients with COPD, even at relatively mild grades. Comparing dyspnea responses across different inclines under conditions of equal oxygen uptake enabled us to isolate the effect of incline from the effect of increased work against gravity.

The main findings are that COPD patients reported significantly greater dyspnea at higher grades, despite identical metabolic load. Breathing frequency and tidal volume were unchanged across grades, whereas dynamic hyperinflation worsened with increasing incline. None of these effects were observed in healthy participants. In addition, the effect of the incline was associated with a statistically significant albeit small change in gait variability, with no other changes in gait kinematics (step time and step length). 

Taken together, these findings demonstrate that walking uphill, in itself, is associated with more severe dyspnea on exertion in COPD patients, even when metabolic load is matched across grades; and they suggest that this effect may be mediated, at least in part, by worsening dynamic hyperinflation.

Exertional dyspnea is the most common symptom of COPD [4]. In an international survey, 68% of COPD patients complained of breathlessness when walking up one flight of stairs [16]. COPD patients often complain that walking uphill greatly exacerbates dyspnea, even when the grade is very mild. In this study we investigated the effect of treadmill grade on dyspnea in patients with mild to very severe COPD. Dyspnea is believed to arise from an imbalance between inspiratory neural drive (IND) to breath and the capacity of the respiratory system to respond [6]. In COPD an appropriate ventilatory response to effort is hampered by airway obstruction. IND during exercise in this patient population is enhanced by both chemical and mechanical stimuli: increased chemo-stimulation due to ventilation-perfusion mismatch, leading to hypoxemia and hypercarbia as well as early metabolic acidosis due to deconditioning and sarcopenia, together with feedback from activated mechanoreceptors in the respiratory muscles and working peripheral muscles [15,17,18]. It appears that dynamic hyperinflation (DH) plays a central role in limiting exercise in COPD across a wide range of severity of airway obstruction [19,20,21]. As ventilatory demand increases during exercise, a combination of increased respiratory rate and expiratory flow limitation force the COPD patient to breath at an abnormally high lung volume, that is, to increase end-expiratory and end-inspiratory lung volumes (EELV and EILV, respectively), in order to satisfy the need for increased expiratory flow [19].

DH is characterized by a progressive decrease of inspiratory capacity (IC) [20], forcing COPD patients to breath on the less compliant portion of the pressure–volume curve, where elastic load and inspiratory work of breathing are greater [4,7,19]. In addition, at higher lung volumes muscle fibers of the diaphragm fiber are shortened. Thus, DH is associated with an increase in the mechanical load on the inspiratory muscles [7], and also places them at a mechanical disadvantage [20]. This increased work and cost of breathing with no further increased in V_T_ may contribute to neuromechanical dissociation, leading to progressive worsening of dyspnea.

A dynamic decrease in IRV, although indirect, may provide key information about neuromechanical dissociation of the respiratory system in COPD. When, as a result of DH, IRV decreases below 0.5–1 L (the “O’Donnell threshold”) [21,22], dyspnea increases sharply, while further V_T_ increase becomes progressively constrained as EILV approaches TLC, suggesting that a significant neuro-mechanical dissociation is causing the increase in dyspnea. In our cohort, IRV at iso-$\dot{V}$O_2_ in the COPD group was an average of 798 ± 336 at 1% grade, 698 ± 325 at 2.5% grade (*p* < 0.002 vs. 1%), and 564 ± 350 at 4% grade (*p* < 0.004 vs. 2.5%, vs. 1%) demonstrating that, at 4% inclines, IRV values approached the O’Donnell threshold.

Our results may have important clinical implications. For example, exercise training is a key component in the management of COPD [23]. Optimal training regimens aim for high intensity exercise [24] above the gas exchange threshold to improve aerobic fitness, thus reducing ventilatory demand at any given task. However, severe dyspnea during exercise training is distressing for COPD patients and impedes effective training. In treadmill exercise, intensity can be increased by increasing grade or by increasing speed. Our results suggest that increasing speed (at 0% grade) may be a preferred strategy for increasing exercise intensity while minimizing increases in dyspnea. Another training strategy might be high-intensity interval training, in which the brevity of the high-intensity period (typically 20 s in a 60 s cycle) may reduce the overall burden of dyspnea, despite the use of significant treadmill grade to attain high-intensity effort. Interventional studies to investigate these possibilities are warranted.

To the best of our knowledge, this is the first study to isolate uphill walking from the metabolic load from the additional work associated with uphill walking, by comparing responses at iso-$\dot{V}$O_2_. Further studies are needed to better understand why walking uphill exacerbates DH in COPD patients and may contribute to a better understanding of the physiology of DH. Walking up an incline may subtly affect the position of the diaphragm, potentially contributing to dynamic hyperinflation [25]. It is also possible that walking uphill may modify afferent signals from the locomotor muscles which may increase IND to the already overloaded respiratory muscles, thus worsening dyspnea by augmenting neuromechanical dissociation. These potential mechanisms warrant further investigation.

### Study Limitations

As previously described [14], the technique used to determine dynamic hyperinflation is challenging and requires a high level of patient cooperation. Therefore, the maneuver was practiced repeatedly by each participant prior to the exercise tests. Misalignment of the tidal flow–volume loop within the maximal loop may result in an inaccurate estimation of exercise IRV. Mechanics and time-constants are different in tidal versus maximal expiration [15], and thoracic gas compression may create an artifact [26]. Despite these limitations, the technique has been widely applied in previous studies [14,19]. In the present study, all exercise tests were supervised by experienced exercise physiologists (RR and AY) with extensive expertise in cardiopulmonary exercise testing in COPD patients. Great care was taken to ensure high-quality measurements. IC maneuvers were practiced prior to the exercise tests with each subject. During the exercise tests, IC maneuvers were repeated twice during each stage. The attending physiologist carefully monitored the spirogram to ensure stability of the tidal volume before instructing the subject to perform the IC maneuver on the next breath. Finally, spirometry was repeated after the test in order to identify exercise-induced bronchodilatation (which in fact was not significant in any of the tests in this study). Nevertheless, some inaccuracy in the measurements may have occurred. In this respect, the progressive effect on respiratory dynamics shown in Figure 3 is reassuring.

Another limitation of our study is the relatively small sample size. This was an exploratory physiological study of significant complexity. We had no previous similar studies on which to base a priori sample size calculation. However post-hoc rank-based effect sizes for the significant primary contrasts were large, with r values ranging from 0.75 to 0.88, suggesting large within-subject effects, although these estimates should be interpreted cautiously because post hoc power is largely determined by the observed *p* value. Due to the small sample size, subgroup analyses to confirm the robustness of the results across subgroups of COPD patients (e.g., GOLD severity or clinical classification) was not performed, which may limit the generalizability of the results to the broader COPD population.

## 5. Conclusions

Walking uphill is associated with greater dyspnea in COPD patients, even at iso-$\dot{V}$O_2_, suggesting that the increased dyspnea cannot be explained simply by the increased metabolic work against gravity. This finding may result from dynamic hyperinflation, which is worse at steeper grades. Further studies are required to more precisely elucidate the underlying mechanism of this phenomenon. 

## Figures and Tables

**Table 1:** Characteristics and lung function of COPD and healthy participants.

	COPD	Healthy Control	*p*-Value
Subjects (n)	14 (F = 4)	9 (F = 2)	
Age (yr)	68 ± 7	67 ± 8	NS
Height (cm)	170 ± 9	171 ± 9	NS
Weight (kg)	75 ± 17	76 ± 11	NS
BMI (kg/m^2^)	26 ± 5	26 ± 2	NS
GOLD A/B/E/unknown	5/7/1/1	-	
GOLD 1/2/3/4	0/5/9/0	-	
COPD Medication (n = 13):			
LAMA	9 (69%)	-	
LABA	12 (92%)	-	
ICS	3 (23%)	-	
LTOT	1 (8%)	-	
FEV_1_ (L)	1.41 ± 0.56	2.99 ± 0.76	*p* < 0.0001
FEV_1_ (% predicted)	49 ± 11	103 ± 12	*p* < 0.0001
FVC (L)	2.86 ± 0.76	3.75 ± 0.96	*p* < 0.039
FVC (% predicted)	80 ± 10	101 ± 10	*p* < 0.0001
FEV_1_/FVC ratio	0.47 ± 0.1	0.80 ± 0.04	*p* < 0.0001
RV (% predicted)	174 ± 33	105 ± 17	*p* < 0.0001
FRC (% predicted)	153 ± 33	104 ± 16	*p* < 0.001
IC (% predicted)	74 ± 15	99 ± 16	*p* < 0.002
TLC (% predicted)	118 ± 21	96 ± 8	*p* < 0.020
RV/TLC (%)	59 ± 6	41 ± 7	*p* < 0.0001
DLCO (% predicted)	64 ± 22	102 ± 10	*p* < 0.001

Values are presented as mean ± SD; *p*-values are for between group comparisons (Mann–Whitney U test). Abbreviations: BMI, Body mass index; GOLD, Global Initiative for Chronic Obstructive Lung Disease; LAMA, Long-acting muscarinic antagonists; LABA, Long-acting beta-agonists; ICS, Inhaled corticosteroids; LTOT, Long term oxygen therapy; FEV_1_, Forced expiratory volume in one second; FVC, Forced vital capacity; RV, Residual volume, FRC, Functional residual capacity; IC, Inspiratory capacity; TLC, Total lung capacity; DLCO, Diffusion capacity for carbon monoxide; NS, Non-significant.

**Table 2:** Measurements at peak of symptom-limited treadmill exercise tests.

	COPD	Healthy	*p*-Value
Peak $\dot{V}$O_2_ (mL/min/kg)	18.1 ± 3.4	24 ± 3.2	*p* = 0.009
Peak $\dot{V}$O_2_ (% predicted)	72 ± 15	83 ± 11	*p* = 0.019
Peak HR (beats/min)	128 ± 19	147 ± 17	*p* = 0.072
Peak HRR (beats/min)	24 ± 18	7 ± 21	
Peak HR (% predicted)	85 ± 15	97 ± 14	*p* = 0.033
$\dot{V}$O_2_/HR (mL/beat)	10 ± 2.9	13 ± 3	*p* = 0.036
RER @ peak	0.92 ± 0.07	1.09 ± 0.1	*p* = 0.0001
Ventilation peak (L/min)	42 ± 12	67 ± 20	*p* = 0.005
Tidal Volume peak (mL)	1238 ± 362	1650 ± 461	*p* = 0.003
B_f_ peak (min-1)	32 ± 8	36 ± 7	NS
Breathing reserve %	18 ± 18	44 ± 13	*p* = 0.001
SpO_2_% peak	93 ± 6	99 ± 1	*p* = 0.0001
$\dot{V}$_E_/$\dot{V}$O_2_ @ GET *	33 ± 6	32 ± 8	NS
$\dot{V}$_E_/$\dot{V}$CO_2_ @ GET *	34 ± 6	31 ± 4	NS
PetCO_2_ @ peak (mmHg)	35 ± 6	37 ± 3	NS

Results at peak exercise were determined for each subject as the highest peak $\dot{V}$O_2_ achieved during the three exercise tests. Values are presented as mean ± SD; *p*-values are for between group comparisons (Mann-Whitney U test). Abbreviations: $\dot{V}$O_2_, Oxygen consumption; HR, Heart rate; HRR, Heart rate reserve; RER, Respiratory exchange ratio; B_f_, Breathing frequency; SpO_2_, Oxygen saturation; $\dot{V}$_E_, Minute ventilation; $\dot{V}$_E_/$\dot{V}$O_2,_ Ventilatory equivalent for oxygen consumption; $\dot{V}$_E_/$\dot{V}$CO_2,_ Ventilatory equivalent for carbon monoxide production, GET, Gas exchange threshold; PetCO_2_, Partial pressure of CO_2_ in end-tidal gas; NS, non-significant; * GET not identified in four patients with FEV_1_ < 50% predicted.

**Table 3:** Physiological parameters and dynamic respiratory mechanics at iso-$\dot{V}$O_2_ at different inclines in COPD subjects.

Grade	1%	2.5%	4%	1% vs. 4%	1% vs. 2.5%	2.5% vs. 4%
$\dot{V}$O_2_ (mL/min)	1156 ± 394 (all grades)					
Dyspnea (Borg scale)	5 ± 2	5 ± 2	7 ± 2	*p* < 0.001	NS	*p* < 0.005
Leg Fatigue (Borg scale)	2 ± 2	2 ± 1	3 ± 1	*p* < 0.003	NS	*p* < 0.004
HR (bpm)	124 ± 22	123 ± 19	122 ± 18	NS	NS	NS
$\dot{V}$O_2_/HR (mL/beat)	10 ± 3	10 ± 3	10 ± 3	NS	NS	NS
Ventilation (L/min)	40 ± 12	40 ± 12	39 ± 10	NS	NS	NS
Tidal Volume (mL)	1201 ± 404	1184 ± 350	1248 ± 397	NS	NS	NS
Breathing Frequency (min-1)	31 ± 10	30 ± 9	31 ± 8	NS	NS	NS
ΔIC (mL)	−232 ± 243	−334 ± 304	−471 ± 274	*p* < 0.001	NS	*p* < 0.002
IRV (mL)	798 ± 336	698 ± 325	564 ± 350	*p* < 0.002	*p* < 0.004	*p* < 0.004
RER	0.90 ± 0.08	0.90 ± 0.07	0.90 ± 0.06	NS	NS	NS
SpO_2_%	94 ± 5	93 ± 6	93 ± 6	NS	NS	NS
$\dot{V}$_E_/$\dot{V}$O_2_	32 ± 5	31 ± 5	32 ± 6	NS	NS	NS
$\dot{V}$_E_/$\dot{V}$CO_2_	35 ± 7	35 ± 7	36 ± 7	NS	NS	NS
PetCO_2_ (mmHg)	34 ± 6	34 ± 7	35 ± 7	NS	NS	NS

Values are presented as mean ± SD. *p*-values are from Dunn’s post-hoc tests, performed only when the Friedman test showed significance. Abbreviations: $\dot{V}$O_2_, Oxygen consumption; HR, Heart rate; ΔIC, Change in inspiratory capacity; IRV, Inspiratory reserve volume; RER, Respiratory exchange ratio; SpO_2_, Oxygen saturation; $\dot{V}$_E_, Minute ventilation; $\dot{V}$_E_/$\dot{V}$O_2,_ Ventilatory equivalent for oxygen consumption; $\dot{V}$_E_/$\dot{V}$CO_2,_ Ventilatory equivalent for carbon dioxide production; PetCO_2_, Partial pressure of CO_2_ in end-tidal gas; NS, Non-significant.

## Data Availability

The original contributions presented in this study are included in the article/Supplementary Material. Further inquiries can be directed to the corresponding author.

## Supplementary Materials

The following supporting information can be downloaded at: https://www.mdpi.com/article/10.3390/jcm15124601/s1, Supplementary S1: Linear interpolation—Method and Example; Figure S1: Graphic demonstration of linear interpolation of Borg dyspnea score to calculate the score at iso-$\dot{V}$O2; Table S1: Physiological parameters and dynamic respiratory mechanics at iso-$\dot{V}$O2 at different inclines in healthy participants; Table S2: Gait analysis.

## Abbreviations

The following abbreviations are used in this manuscript:

BR	Breathing reserve
COPD	Chronic obstructive pulmonary disease
CPET	Cardiopulmonary exercise testing
DLCO	Diffusing capacity for carbon monoxide
EELV	End expiratory lung volume
EILV	End inspiratory lung volume
EVL	Expiratory ventilatory limitation
Ex-tFVL	Tidal flow-volume loops during exercise
FEV1	Forced expiratory volume in one second
FRC	Functional residual capacity
FVC	Forced vital capacity
GET	Gas exchange threshold
IC	Inspiratory capacity
IRV	Inspiratory reserve volume
HR	Heart rate
MaxFVL	Maximal flow-volume loop
MVV	Maximal voluntary ventilation
RER	Respiratory exchange ratio
RPE	Rating of perceived exertion
RV	Residual volume
SpO_2_	Oxygen saturation
VT	Tidal volume
$\dot{V}$CO_2_	Carbon monoxide output
$\dot{V}$E	Minute ventilation
$\dot{V}$O_2_	Oxygen uptake

## References

[1] O’Donnell D.E., Laveneziana P. (2007). Dyspnea and activity limitation in COPD: Mechanical factors. COPD J. Chronic Obstr. Pulm. Dis..

[2] O’DONNELL D.E., Lam M., Webb K.A. (1998). Measurement of symptoms, lung hyperinflation, and endurance during exercise in chronic obstructive pulmonary disease. Am. J. Respir. Crit. Care Med..

[3] O’Donnell D.E. (2001). Ventilatory limitations in chronic obstructive pulmonary disease. Med. Sci. Sports Exerc..

[4] Stickland M.K., Neder J.A., Guenette J.A., O’Donnell D.E., Jensen D. (2022). Using Cardiopulmonary Exercise Testing to Understand Dyspnea and Exercise Intolerance in Respiratory Disease. Chest.

[5] Ofir D., Laveneziana P., Webb K.A., Lam Y.M., O’Donnell D.E. (2008). Mechanisms of dyspnea during cycle exercise in symptomatic patients with GOLD stage I chronic obstructive pulmonary disease. Am. J. Respir. Crit. Care Med..

[6] James M.D., Milne K.M., Phillips D.B., Neder J.A., O’Donnell D.E. (2020). Dyspnea and Exercise Limitation in Mild COPD: The Value of CPET. Front. Med..

[7] Sietsema K.E., Rossiter H.B. (2023). Exercise Physiology and Cardiopulmonary Exercise Testing. Semin. Respir. Crit. Care Med..

[8] Johnson B.D., Weisman I.M., Zeballos R.J., Beck K.C. (1999). Emerging concepts in the evaluation of ventilatory limitation during exercise: The exercise tidal flow-volume loop. Chest.

[9] Aitken C.R., Walsh J.R., Sabapathy S., Adams L., Morris N.R., Stewart G.M. (2022). Optimising the Dyspnoea Challenge: Exertional dyspnoea responses to changing treadmill gradients. Respir. Physiol. Neurobiol..

[10] Quanjer P.H., Stanojevic S., Cole T.J., Baur X., Hall G.L., Culver B.H., Enright P.L., Hankinson J.L., Ip M.S., Zheng J. (2012). Multi-ethnic reference values for spirometry for the 3-95-yr age range: The global lung function 2012 equations. Eur. Respir. J..

[11] Ross R.M. (2003). ATS/ACCP statement on cardiopulmonary exercise testing. Am. J. Respir. Crit. Care Med..

[12] Kaminsky L.A., Arena R., Myers J., Peterman J.E., Bonikowske A.R., Harber M.P., Medina Inojosa J.R., Lavie C.J., Squires R.W. (2022). Updated Reference Standards for Cardiorespiratory Fitness Measured with Cardiopulmonary Exercise Testing: Data from the Fitness Registry and the Importance of Exercise National Database (FRIEND). Mayo Clin. Proc..

[13] Wasserman K. (1987). Determinants and detection of anaerobic threshold and consequences of exercise above it. Circulation.

[14] Reuveny R., Vilozni D., Dagan A., Ashkenazi M., Velner A., Segel M.J. (2022). The role of inspiratory capacity and tidal flow in diagnosing exercise ventilatory limitation in Cystic Fibrosis. Respir. Med..

[15] Guenette J.A., Chin R.C., Cory J.M., Webb K.A., O’Donnell D.E. (2013). Inspiratory Capacity during Exercise: Measurement, Analysis, and Interpretation. Pulm. Med..

[16] Rennard S., Decramer M., Calverley P.M., Pride N.B., Soriano J.B., Vermeire P.A., Vestbo J. (2002). Impact of COPD in North America and Europe in 2000: Subjects’ perspective of Confronting COPD International Survey. Eur. Respir. J..

[17] Neder J.A., Arbex F.F., Alencar M.C., O’Donnell C.D., Cory J., Webb K.A., O’Donnell D.E. (2015). Exercise ventilatory inefficiency in mild to end-stage COPD. Eur. Respir. J..

[18] O’Donnell D.E., Laveneziana P., Neder J.A. (2021). Editorial: Clinical Cardiopulmonary Exercise Testing. Front. Physiol..

[19] Milne K.M., Domnik N.J., Phillips D.B., James M.D., Vincent S.G., Neder J.A., O’Donnell D.E. (2020). Evaluation of Dynamic Respiratory Mechanical Abnormalities During Conventional CPET. Front. Med..

[20] O’Donnell D.E., Ora J., Webb K.A., Laveneziana P., Jensen D. (2009). Mechanisms of activity-related dyspnea in pulmonary diseases. Respir. Physiol. Neurobiol..

[21] Chin R.C., Guenette J.A., Cheng S., Raghavan N., Amornputtisathaporn N., Cortes-Telles A., Webb K.A., O’Donnell D.E. (2013). Does the respiratory system limit exercise in mild chronic obstructive pulmonary disease?. Am. J. Respir. Crit. Care Med..

[22] Casaburi R., Rennard S.I. (2015). Exercise limitation in chronic obstructive pulmonary disease. The O’Donnell threshold. Am. J. Respir. Crit. Care Med..

[23] Troosters T., Casaburi R., Gosselink R., Decramer M. (2005). Pulmonary rehabilitation in chronic obstructive pulmonary disease. Am. J. Respir. Crit. Care Med..

[24] Emtner M., Porszasz J., Burns M., Somfay A., Casaburi R. (2003). Benefits of supplemental oxygen in exercise training in nonhypoxemic chronic obstructive pulmonary disease patients. Am. J. Respir. Crit. Care Med..

[25] Cao Y., Li P., Wang Y., Liu X., Wu W. (2022). Diaphragm Dysfunction and Rehabilitation Strategy in Patients with Chronic Obstructive Pulmonary Disease. Front. Physiol..

[26] Guenette J.A., Dominelli P.B., Reeve S.S., Durkin C.M., Eves N.D., Sheel A.W. (2010). Effect of thoracic gas compression and bronchodilation on the assessment of expiratory flow limitation during exercise in healthy humans. Respir. Physiol. Neurobiol..

