# Supplementary material for: Walking Uphill Aggravates Dyspnea and Dynamic Hyperinflation at Equivalent Oxygen Uptake in COPD Patients
Source: J Clin Med. 2026 Jun 13;15(12):4601. doi: 10.3390/jcm15124601 (PMC13301334; doi:10.3390/jcm15124601)
Supplement: Supplementary file 1 [file jcm-15-04601-s001.zip › jcm-4326418-supplementary.pdf]

Supplement S1: Linear interpolation – Method and Example

To compare the three tests under a uniform metabolic load, parameters were evaluated at the same $\dot{V}O_2$ level (iso-$\dot{V}O_2$). For each subject, the lowest measured $\dot{V}O_2$ peak across the three tests was used as the point of comparison (iso-$\dot{V}O_2$). Parameter values at iso-$\dot{V}O_2$ were calculated from the measured values by linear interpolation.

For example: COPD patient No. 9—$\dot{V}O_2$ peak at treadmill grades of 1%, 2.5%, and 4% were 1440, 1400, and 1120 ml/min, respectively. Therefore, iso-$\dot{V}O_2$ was selected as the lowest of the three-peak $\dot{V}O_2$ values—1100 ml/min. Next, we used a custom Microsoft Excel™-based calculator to linearly interpolate the Borg dyspnea score at iso-$\dot{V}O_2$ for each of the three tests, as shown in Figure 1 for a 2.5% treadmill grade. The same procedure was repeated for each subject.

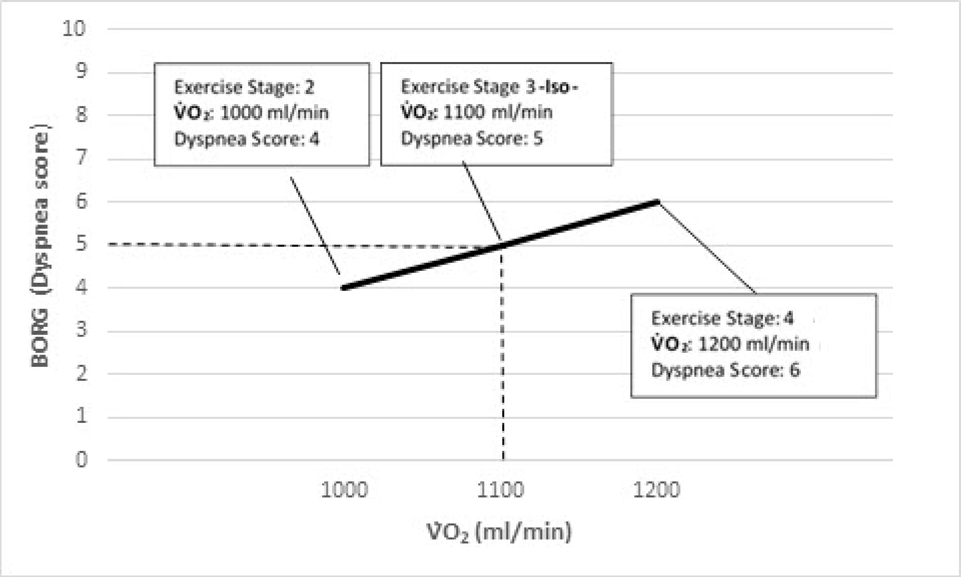

Figure S1. Graphic demonstration of linear interpolation of Borg dyspnea score to calculate the score at iso-$\dot{V}O_2$. After iso-$\dot{V}O_2$ was calculated, Borg score and $\dot{V}O_2$ for the stages before and after iso-$\dot{V}O_2$ were plotted, then connected by a straight line (solid line). Thus, Borg score at iso-$\dot{V}O_2$ could be determined (dashed lines).

Supplement Table S1 - Physiological parameters and dynamic respiratory mechanics at iso-$\dot{V}O_2$ at different inclines in healthy participants

	**Grade 1%**	**Grade 3%**	**Grade 5%**
$\dot{V}O_2$ (ml/min)		1081±375	
Dyspnea (Borg scale)	2±1	2±2	2±1
Leg Fatigue (Borg scale)	2±2	3±2	3±2
HR (bpm)	136±13	133±17	135±14
$\dot{V}O_2$/HR (ml/beat)	13±3	12±3	12±3
Ventilation (l/min)	57±20	55±16	56±17
Tidal Volume (ml)	1368±640	1289±614	1300±568
Breathing Frequency (min-1)	36±9	33±7	33±6
ΔIC (ml)	164±106	148±106	210±166
IRV (ml)	1770±841	1758±841	1811±933
RER	1.0±0.1	1.0±0.1	1.0±0.1
SPO_2 %	99±0.5	98±0.6	99±0.5
$\dot{V}_E$/ $\dot{V}O_2$	30±6	30±7	30±7
$\dot{V}_E$/ $\dot{V}CO_2$	31±5	30±4	30±4
$PetCO_2$ (mmHg)	36±3	36±3	36±3

Values are presented as mean ± SD; all comparisons between grades were not statistically significant.

Supplement Table S2: Gait analysis

	COPD	Healthy

Incline	1.0%	2.5%	4.0%	1.0%	3.0%	5.0%
Treadmill Iso-Speed (km/h)		4.0 (2.3-6)			7.0 (5.5-7.0)	
Step length (cm)	55.2 (49.8-69.3)	68.9 (51.8-79.1)	68.1 (55.5-72.9)	84.7 (81.0-88.9)	82.6 (71.7-85.5)	87.7 (77.8-87.9)
CV Step length (%)	4.0 (2.8-6.1)	4.6 (4.1-5.9)	4.4 (3.3-7.5)	3.4 (2.0-4.3)	4.6 (3.9-4.8)	4.3 (4.0-4.7)
Step time (sec)	0.57 (0.49-0.63)	0.58 (0.50-0.62)	0.58 (0.52-0.62)	0.45 (0.43-0.46)	0.47 (0.44-0.51)	0.46 (0.45-0.47)
CV Step time (%)	2.7 (2.5-4.7)	4.1 (2.7-4.6)	3.6 (3.0-5.1)	2.4 (2.2-3.3)	3.3 (2.7-3.6)	3.4 (3.0-4.0)

Data are presented as median (interquartile range). Treadmill Iso Speed: the highest common speed that was present in all incline conditions; CV: Coefficient of variation.
